# Prevalence and factors associated with resilience in Peruvian adolescent schoolchildren during the early post-pandemic context: a cross-sectional study

**DOI:** 10.3389/fpsyt.2025.1609190

**Published:** 2025-07-25

**Authors:** Mario J. Valladares-Garrido, Luis E. Cueva-Cañola, Pamela Grados-Espinoza, Luz A. Aguilar-Manay, Jassmin Santin Vásquez, Danai Valladares-Garrido, César J. Pereira-Victorio, Víctor J. Vera-Ponce

**Affiliations:** ^1^ Escuela de Medicina Humana, Universidad Señor de Sipán, Chiclayo, Peru; ^2^ Facultad de Ciencias de la Salud, Universidad Nacional de Piura, Piura, Peru; ^3^ Red Latinoamericana de Medicina en la Altitud e Investigación (REDLAMAI), Pasco, Peru; ^4^ Facultad de Medicina, Universidad San Martin de Porres, Chiclayo, Peru; ^5^ Escuela de Medicina, Universidad Cesar Vallejo, Piura, Peru; ^6^ Oficina de Salud Ocupacional, Hospital Santa Rosa, Piura, Peru; ^7^ Facultad de Medicina, Universidad Continental, Lima, Peru; ^8^ Departamento de Ciencias Médicas, Facultad de Ciencias de la Salud, Universidad Castilla La Mancha, Talavera de la Reina, Spain; ^9^ Instituto de Investigación de Enfermedades Tropicales, Universidad Nacional Toribio Rodríguez de Mendoza de Amazonas, Chachapoyas, Amazonas, Peru; ^10^ Facultad de Medicina (FAMED), Universidad Nacional Toribio Rodríguez de Mendoza de Amazonas (UNTRM), Amazonas, Peru

**Keywords:** resilience, psychological, adolescent, mental health, risk factors, schools, Peru

## Abstract

**Introduction:**

Resilience is a fundamental protective factor during adolescence, as it enables young people to cope with adversity and reduce the risk of mental health disorders. However, evidence on the prevalence and associated factors of resilience in adolescents remains limited in Latin America, particularly in school settings and the post-pandemic context. This study aimed to estimate the prevalence and associated factors of resilience among adolescents from five educational institutions in northern Peru.

**Methods:**

An analytical cross-sectional study was conducted between September and December 2022, in the post-pandemic period, among 1,307 adolescents from five schools in the Lambayeque region, Peru. Data were collected using a self-administered digital questionnaire during school hours. Adolescents who regularly attended classes and completed all required items of the CD-RISC-10 scale were included. This secondary analysis of data used data from a primary study on adolescent mental health. The dependent variable was resilience, measured using the abbreviated CD-RISC-10 scale, and categorized as low (0–29 points) or high (≥30 points). Independent variables included sociodemographic, family, academic, and behavioral factors. Descriptive, bivariate, and multivariate analyses were conducted using generalized linear models (Poisson family) with robust variance, and prevalence ratios (PR) with 95% confidence intervals (95% CI) were reported.

**Results:**

The prevalence of high resilience was 17.3% (95% CI: 15.28–19.45). In multivariate analysis, frequent closeness with friends (PR: 1.93) and with relatives (PR: 1.47) were associated with higher resilience. Conversely, residing in urban areas (PR: 0.82), belonging to a non-Catholic religion (PR: 0.68), failing a course (PR: 0.78), cigarette use (PR: 0.46), and excessive internet use (>11 hours/day) (PR: 0.87) were associated with lower resilience.

**Conclusions:**

These findings underscore the importance of social and family support in fostering resilience during adolescence during the early post-pandemic context. They also highlight the need for interventions to address modifiable risk factors, including smoking, excessive screen time, and poor academic performance. The implementation of mentoring and psychological counseling programs within schools, alongside strategies to promote resilience in educational and family environments, is strongly recommended.

## Introduction

Adolescence is a critical developmental and transitional period, characterized by brain and body maturation, increased socialization, and the shift toward independence (1). Various studies have shown that adolescents are more vulnerable to traumatic and stressful events, making them more likely to develop mental health disorders when exposed to such circumstances, which may compromise their development and future (1). Since December 2019, Coronavirus Disease 2019 (COVID-19) spread globally, becoming a pandemic that affected all continents (2). The pandemic and resulting lockdowns produced mental health consequences in adolescents, including chronic and acute stress (1, 3), concern for their families, unanticipated bereavement, suspension of in-person schooling, home confinement, and increased access to the Internet and social networks (2). Nevertheless, many adolescents exposed to disasters or epidemics manage to cope with such experiences, demonstrating resilience and effective coping strategies (2).

Resilience is not a fixed trait, but a dynamic, context-sensitive, and culturally embedded process that evolves in response to adversity (4). It has been defined as the ability to recover from negative emotional experiences and the capacity to maintain competent functioning in the face of major life stressors (5). It is also described as the process of adapting well when faced with adversity, trauma, or significant sources of stress, which implies that resilience can only be observed in response to such challenges (5, 6). In adolescence, a stage marked by intense biological, emotional, and social changes—resilience involves overcoming negative experiences through constructive self-improvement and adaptive strategies (7). Developing positive coping mechanisms during this period is crucial for addressing academic demands and acquiring essential social competencies (7).

To conceptualize resilience more comprehensively, this study draws on Bronfenbrenner’s ecological systems theory, which posits that adolescent development is influenced by multiple, nested systems, ranging from individual-level characteristics to family dynamics, peer relationships, and broader societal influences. In this framework, resilience emerges through the dynamic interplay between risk and protective factors across these ecological layers. Thus, examining resilience requires considering intrapersonal traits (e.g., self-esteem, coping), family functioning (e.g., parenting, cohesion), and environmental supports (e.g., school climate, peer connections) (8–10). This framework is especially relevant in Latin American countries such as Peru, where cultural values—such as collectivism, strong familial bonds, religious identity, and community interdependence—may influence how resilience is expressed, perceived, and cultivated among adolescents.

Furthermore, the literature reports wide-ranging prevalence estimates of high resilience among adolescents across countries, suggesting the need for culturally grounded assessments and interpretations. Studies from Nepal, Nigeria, Australia, Iran, and Austria report prevalences ranging from 6.3% to 73% (11–15), while Latin American studies show 27.7% in Colombia and up to 92% in Brazil (16, 17). In Peru, it is reported that between 41.7-70.2% of Peruvian adolescents have a high level of resilience (18–20). There are factors associated with a high level of resilience in adolescents such as individual (aspirations for the future such as success, money, employment, among others (21), self-esteem, autonomy, facing their doubts and emotions (22), family (family communication (23), parenting styles (24), cohesion, warmth and low level of discord (22), social (social support (23), friendship (21), psychological (hope, sense of coherence (4), courageous coping (23) and educational-related to their school (school climate) (24, 25). There is evidence that adolescents with greater resilience resources invest more time in learning tasks, are more participative and perform better academically than those with lower resilience resources (7, 26). Most resilience studies conducted in adolescents have identified associations with school performance (27), social skills (28), mental health disorders (29) or focus on evaluating universal resilience interventions, as reported in a systematic review (30).

However, there is still inconclusive evidence regarding resilience and its associated factors in adolescents, due to several limitations. First, there are few studies that focus on assessing resilience and its associated factors in adolescents in a school setting (31), including in Latin America (21) and Peru (18–20). Second, previous studies have information bias since they have not measured important variables such as internet and social network use, substance use (29), religion (32), family type (28), mental history (12), family mental history (32), having sought mental help due to the pandemic (18). Third, findings from previous studies lack representativeness because they have not evaluated multiple secondary school sites (20, 33). Fourth, previous evidence has small sample sizes; therefore, low statistical power. Fifth, previous studies have not robustly evaluated the factors associated with resilience through multivariate analysis, much less bivariate analysis (7, 12) since they are descriptive (18, 20, 32). Finally, few studies have examined resilience in adolescents in a post-pandemic context (34, 35).

Therefore, the aim of this study was to evaluate the prevalence and associated factors of resilience among adolescents from five schools in northern Peru, during the early post-pandemic period.

## Methods

### Study design

We conducted an analytical cross-sectional study based on secondary analysis of data collected from adolescents attending five educational institutions in the Lambayeque region of Peru. This aim was to estimate the prevalence and factors associated with resilience in adolescents. The original database came from a primary study that investigated the relationship between acne and mental health outcomes.

Although the original study focused on acne, its protocol included standardized measures of psychological variables, including resilience, assessed using the Connor-Davidson Resilience Scale–10-item versión (CD-RISC-10) questionnaire. This was based on the recognition that resilience is a key protective factor in adolescence, particularly in the face of stressors such as visible dermatological conditions, social isolation during the COVID-19 pandemic, and academic challenges. Therefore, this secondary analysis was designed specifically to explore resilience, leveraging the comprehensive dataset collected in the original study. This methodological approach is aligned with current ethical and scientific standards for secondary data analysis and is consistent with the overarching goal of advancing adolescent mental health research in the post-pandemic context.

### Population and sample

The target population consisted of 1,972 students enrolled in the five selected educational centers in the Lambayeque region between September and December 2022, during the early post-pandemic context.

In the primary study, the final sample included 1,442 adolescents who regularly attended school and completed the main research instruments. Participants were excluded if they lacked parental consent, declined assent, or submitted incomplete responses.

In the present secondary analysis, an additional data cleaning process was conducted to ensure the integrity of the outcome measurement. Specifically, 135 records were excluded because participants did not fully complete the abbreviated Connor-Davidson Resilience Scale (CD-RISC-10), which served as the main outcome variable in this study. A non-probability (convenience) sampling approach was used. Out of the 1,972 students enrolled in the five selected schools, a total of 1,307 adolescents were included in the final analytic sample. This corresponds to a final participation rate of 66.3%, calculated as the proportion of students who completed the CD-RISC-10 items relative to the total enrolled population.

A *post hoc* power analysis indicated that the final sample size provided sufficient statistical power to detect key associations. For example, the study had 93.1% power to detect differences in resilience based on family closeness and 77.8% power for the association with course failure, using the observed proportions and subgroup sample sizes.

### Procedures

Data collection took place between September and December 2022 in five educational institutions in the Lambayeque region of Peru. By that time, Peru had exited its fourth wave of COVID-19, most public health restrictions were lifted, and in-person schooling had resumed. This post-pandemic context is important for interpreting the psychosocial conditions and resilience levels reported in this study.

A self-administered digital questionnaire was used, developed using the REDCap (Research Electronic Data Capture) platform. It was designed to assess various sociodemographic factors, mental health history, interpersonal relationships, academic performance, and lifestyle behaviors.

Before implementation, the research team coordinated with school administrators to ensure logistical feasibility. Participants were informed about the study’s objectives and were given informed consent forms for their parents or legal guardians to sign, along with assent forms for the adolescents. Only those who completed both consent and assent were included.

The questionnaire was administered during school hours in classrooms designated by school authorities, under the supervision of trained teachers and research staff. Standardized instructions were provided, and any doubts were resolved before starting. Completion time ranged from 25 to 30 minutes.

To ensure confidentiality and anonymity, the questionnaire did not include any personal identifiers. After collection, data were digitized, cleaned, and verified for consistency and completeness prior to analysis.

### Variables and instruments

The dependent variable in the study was resilience, which was operationalized using the score from the abbreviated version of the Connor-Davidson Resilience Scale (CD-RISC-10). Resilience scores were categorized into two levels: low (0–29 points) and high (30 or more points), based on the sum of the instrument’s items (36).

The independent variables included in the study covered sociodemographic dimensions, mental health history, life habits and family experiences.

Among the sociodemographic variables, age was categorized into early, middle and late adolescence based on the World Health Organization’s developmental stages: early adolescence (10–13 years), middle adolescence (14–16 years), and late adolescence (17–19 years); sex (male or female); type of educational institution (national or private), school grade (first to fifth year of secondary school) and residence area (rural, urban or peri- urban). The number of households members was grouped as 1- to 5, 6–10 or 11-15. Religious affiliation was recorded as none, Catholic or other beliefs.

Regarding mental health history, the presence of personal and family diagnoses was considered, distinguishing between those with and without a reported history. Nutritional status was assessed using body mass index (BMI), categorized as underweight, normal weight, overweight, or obese.

Interpersonal relationships were evaluated based on the frequency of closeness with family and friends, classified as infrequent, frequent, or very frequent. Academic performance was self-reported and categorized as very poor, poor, fair, good, or very good. Additionally, course failure was assessed by determining whether the student had failed at least one subject (yes/no).

In terms of behavioral and personal factors, romantic relationship status was included (having or not having a partner). Substance use was assessed through cigarette use (yes/no) and alcohol consumption, categorized as never, monthly (2–4 times), or frequent (2–4 times per week).

We also assessed whether participants had sought mental health support at any point (yes/no). Daily use of electronic devices, specifically internet and television, was recorded and categorized as 1–5, 6–10, or 11–15 hours per day.

Finally, variables related to the COVID-19 pandemic included whether a family member had been hospitalized due to COVID-19 and whether the participant experienced the loss of a family member from the disease (both coded as yes/no).

Abbreviated CD-RISC: The Connor and Davidson Resilience Scale (CD-RISC) assesses resilience as a very broad domain which should be addressed by 5 dimensions (personal competence, tolerance of negative affect, positive acceptance of change, control and spiritual influences) (37), has 10 items and a 5-point Likert-type scale (4: always, 3: almost always, 2: sometimes, 1: rarely and 0: never) (38, 39). The final score of the questionnaire summed the responses obtained in each item (range 0-40) and the highest scores indicated the highest level of resilience (40). This scale has been validated in adolescent mothers in Peru, adolescents in Colombia, children in China, schoolchildren in Germany and university students in Spain, obtaining Cronbach’s alpha values of 0.85, 0.88, 0.86, 0.81 and 0.85, respectively, making it an optimal, reliable and valid tool (40–44). It should also be noted that this instrument was used in studies during the COVID-19 pandemic in adolescents (45–47). For this study, the Spanish version of the CD-RISC-10 validated and adapted by Notario-Pacheco et al. was used (40).

The cut-off point of ≥30 for high resilience on the CD-RISC-10 was selected based on its use in previous literature and its contextual relevance. Although the CD-RISC-10 does not have a universally established threshold, especially in adolescent populations, this value has been applied in Peruvian research during the COVID-19 pandemic. For example, Leiva León (2021) used this cut-off to dichotomize resilience levels among healthcare workers in Peru, demonstrating its practical applicability in local populations exposed to pandemic-related stress (36). Additionally, this cut-off point was previously applied in different Peruvian studies conducted during the COVID-19 pandemic, where resilience was dichotomized using the CD-RISC-10 with a threshold of ≥30 (48–50).

Moreover, international studies involving both adults and adolescents have employed alternative classification strategies, such as percentile- or tercile-based groupings. Scali et al. divided resilience scores into three categories, with the highest tercile corresponding to scores above 29 (51), while Notario-Pacheco et al. used quartile-based classifications among Spanish adolescents (40). Although these studies did not propose ≥30 as a universal threshold, their categorizations support the interpretation of scores ≥30 as indicative of high resilience.

To ensure the robustness of our findings, we conducted a sensitivity analysis using an alternative threshold of ≥32, which corresponds to a more conservative definition of high resilience. The main associations remained stable—specifically, urban residence, non-Catholic religious affiliation, frequent and very frequent closeness with friends, frequent closeness with family during COVID-19, and cigarette use continued to be significantly associated with resilience. Additionally, mental health support-seeking emerged as a new protective factor. Conversely, academic failure and the use of the internet and television were no longer associated with the adjusted models. These results reinforce the stability and internal validity of our findings across different classification criteria. Finally, in our sample, the internal consistency of the CD-RISC-10 was excellent, with a Cronbach’s alpha of 0.94. This confirms the scale’s reliability for measuring resilience among Peruvian adolescents in a post-pandemic school context.

### Analysis plan

Data were analyzed using Stata version 17.0 software (StataCorp LP, College Station, TX, USA).

First, a descriptive analysis was conducted. Categorical variables were summarized using frequencies and percentages, while continuous variables, such as age, were described using appropriate measures of central tendency and dispersion, based on the distribution’s normality.

To explore associations between resilience and the variables of interest, a bivariate analysis was performed using the chi-square test of independence, after verifying that expected frequencies met the test assumptions. Subsequently, unadjusted and adjusted regression models were used to identify factors associated with resilience.

Generalized linear models (GLM) with a Poisson distribution, robust variance and logarithmic link function were applied, considering the school as the cluster unit. Prevalence ratios (PR) were calculated with their respective 95% confidence intervals (95% CI). In the adjusted model, those variables that showed a significant association in the univariate analysis were included (p < 0.05) and the presence of collinearity between the independent variables in the final model was evaluated. To assess potential multicollinearity among the independent variables included in the multivariate model, we calculated Variance Inflation Factors (VIF). The VIF values for all covariates ranged from 1.03 to 1.66, with a mean VIF of 1.29. These values are well below the commonly accepted threshold of 5.0, indicating no evidence of multicollinearity.

Although the outcome variable (resilience) was binary, we chose to use generalized linear models with a Poisson distribution and robust variance rather than logistic regression. This approach is recommended in cross-sectional studies when the outcome is common (i.e., prevalence >10%) because it allows direct estimation of PR instead of odds ratios (OR), which can overestimate the strength of association. The Poisson regression with robust variance thus provides more interpretable and accurate estimates of association in our epidemiological context.

### Ethical aspects

The primary study was approved by the Ethics Committee of the Universidad San Martín de Porres, Lima, Peru, ensuring compliance with the ethical principles established for research involving human subjects. The confidentiality of the participants was protected through the use of anonymous questionnaires. Likewise, the assent of the adolescents and the informed consent of the parents or legal guardians were obtained, guaranteeing voluntary and ethical participation in the research.

Although the present manuscript is based on secondary analysis, the data were originally collected as part of a primary study that involved human participants. That primary study received approval from the Ethics Committee of the Universidad San Martín de Porres, Lima, Peru, and followed all ethical procedures, including informed consent and assent. The current secondary analysis used anonymized data with no possibility of identifying participants. We clarify this point to resolve any potential discrepancy with automatically generated statements in the submission system regarding the involvement of human subjects.

## Results

### Socioeducational characteristics of the adolescents

A total of 1,307 adolescents were included with a mean age of 14.63 ± 1.40 years. Most of the participants were in the middle stage of adolescence (69.2%) and were female (54.3%). Regarding mental health history, 9.6% reported a personal diagnosis of mental illness and 14.8% had a family history. Regarding nutritional status, 63.1% had a normal BMI. On interpersonal relationships, 45.2% reported frequent closeness with family members and 47.0% with friends. Regarding academic performance, 41.2% indicated good performance. On the use of technologies, 61.7% reported using the Internet between 1 to 5 hours per day. During the COVID-19 pandemic, 50.3% had a family member hospitalized and 44.3% lost a family member. Finally, in terms of resilience, 82.7% presented a low level (**Table 1**).

**Table 1:** Socio-demographic, academic, family and behavioral characteristics of the adolescent sample (n=1307).

Characteristics	N (%)
Age (years)*	14.63 ± 1.40
Adolescent developmental stage	
Early	296 (22.7)
Middle	905 (69.2)
Late	106 (8.1)
Sex	
Male	598 (45.8)
Female	709 (54.3)
Type of school	
Public	851 (65.1)
Private	456 (34.9)
School grade	
First	217 (16.6)
Second	298 (22.8)
Third	264 (20.2)
Fourth	284 (21.7)
Fifth	244 (18.7)
Residence area	
Rural	185 (14.2)
Urban	1087 (83.2)
Peri-urban	35 (2.7)
Number of family members (categorized)	
1 to 5	784 (60.0)
6 to 10	474 (36.3)
11 to 15	49 (3.8)
Religion	
None	305 (23.3)
Catholic	739 (56.5)
Non-Catholic	263 (20.1)
Personal mental health history	
No	1182 (90.4)
Yes	125 (9.6)
Family mental health history	
No	1114 (85.2)
Yes	193 (14.8)
Categorized BMI	
Underweight	276 (21.1)
Normal	824 (63.1)
Overweight	166 (12.7)
Obesity	41 (3.1)
Closeness with family members	
Infrequent	404 (30.9)
Frequent	591 (45.2)
Very frequent	312 (23.9)
Closeness with friends	
Infrequent	314 (24.0)
Frequent	614 (47.0)
Very frequent	379 (29.0)
Academic performance	
Very poor	29 (2.2)
Poor	47 (3.6)
Fair	524 (40.1)
Good	543 (41.2)
Very good	164 (12.6)
Failed a course during school years	
No	710 (54.3)
Yes	597 (45.7)
In a romantic relationship	
No	492 (37.6)
Yes	815 (62.4)
Cigarette use	
No	1228 (94.0)
Yes	79 (6.0)
Alcohol use	
Never	1022 (78.2)
Monthly (2 to 4 times)	246 (18.8)
2 to 4 times per week	39 (3.0)
Sought mental health support	
No	1033 (79.0)
Yes	274 (21.0)
Daily internet use	
1 to 5 hours	807 (61.7)
6 to 10 hours	296 (22.7)
11 to 15 hours	204 (15.6)
Daily television use	
1 to 5 hours	1207 (92.4)
6 to 10 hours	71 (5.4)
11 to 15 hours	29 (2.2)
Family member hospitalized due to COVID-19	
No	649 (49.7)
Yes	658 (50.3)
Family member deceased due to COVID-19	
No	728 (55.7)
Yes	579 (44.3)
Resilience	
Low	1081 (82.7)
High	226 (17.3)

*Mean ± standard deviation.

BMI, Body Mass Index.

### Resilience in adolescents

**Figure 1:**
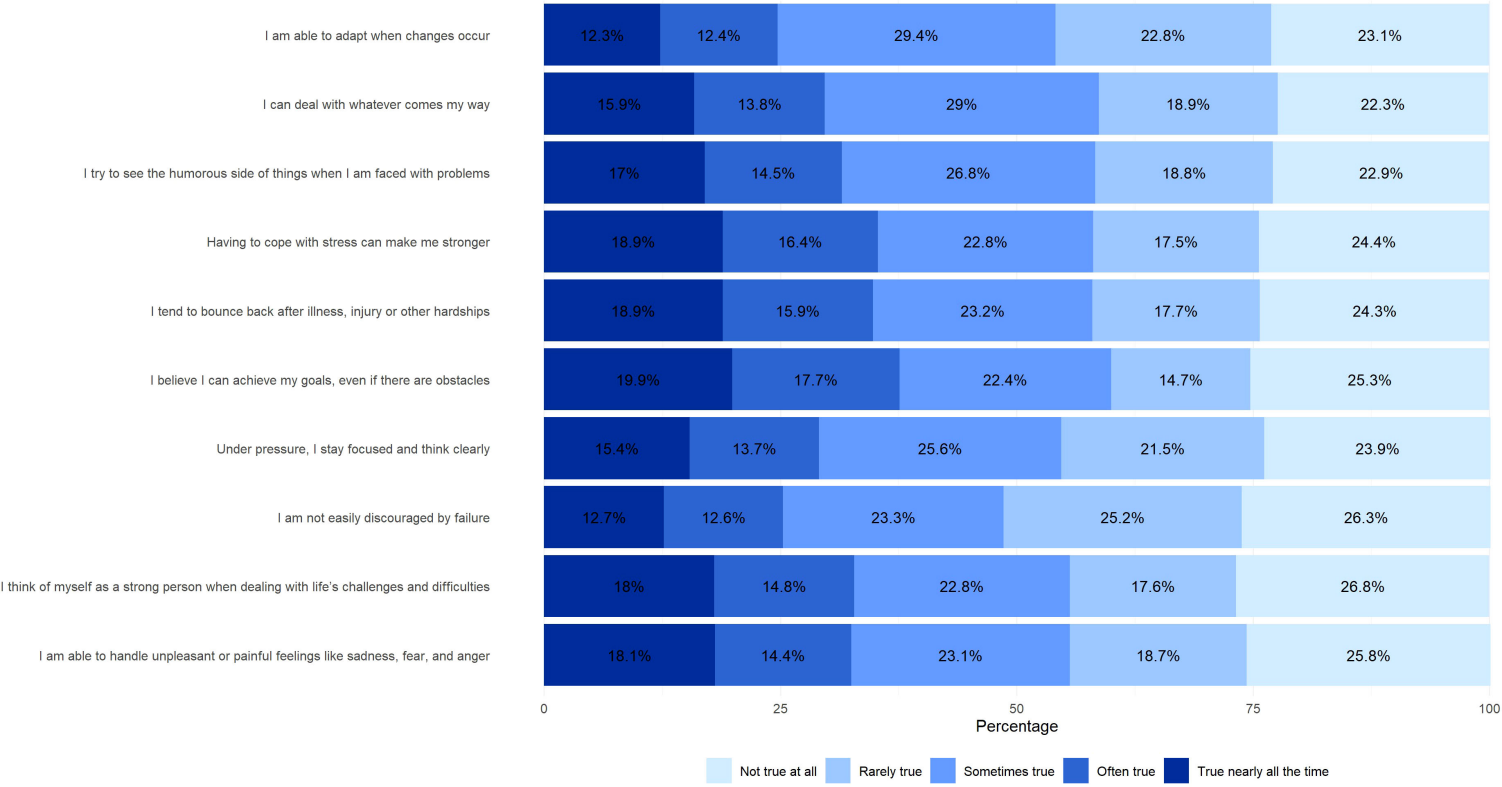
The percentage distribution of responses to the items of the CD-RISC-10 scale.

The prevalence of low level of resilience was 17.3% (95%CI: 15.28 - 19.45). **Figure 1** shows the percentage distribution of responses to the items of the CD-RISC-10 scale, used to assess resilience in adolescents from five schools in northern Peru. A total of 18.9% of respondents indicated that dealing with stress makes them stronger, while 18.9% reported that they usually recover after an illness, injury or difficulty. Likewise, 19.9% of adolescents stated that they believe in their ability to achieve their goals despite obstacles.

### Factors associated with resilience in bivariate analysis

In bivariate analysis, several variables showed significant associations with high resilience. Female adolescents reported slightly higher resilience than males. Greater emotional closeness with family and friends was linked to higher resilience. Academic performance also played a key role: students with better performance and those who had not failed courses showed greater resilience. Conversely, cigarette smoking was associated with lower resilience (**Table 2**).

**Table 2:** Factors associated with resilience in bivariate analysis among adolescents.

Characteristics	Resilience		p*
	Low (n=1081)	High (n=226)	
	n (%)	n (%)	
Adolescent developmental stage			0.402
Early	251 (84.8)	45 (15.2)	
Middle	740 (81.8)	165 (18.2)	
Late	90 (84.9)	16 (15.1)	
Sex			0.049
Male	508 (85.0)	90 (15.1)	
Female	573 (80.8)	136 (19.2)	
Type of school			0.860
Public	705 (82.4)	146 (17.2)	
Private	376 (82.5)	80 (17.5)	
School grade			0.345
First	184 (84.8)	33 (15.2)	
Second	252 (84.6)	46 (15.4)	
Third	217 (82.2)	47 (17.8)	
Fourth	224 (78.9)	60 (21.1)	
Fifth	204 (83.61)	40 (17.3)	
Residence area			
Rural	148 (80.0)	37 (20.0)	0.364
Urban	906 (83.4)	181 (16.7)	
Peri-urban	27 (77.1)	8 (22.9)	
Number of family members (categorized)			0.983
1 to 5	648 (82.7)	136 (17.4)	
6 to 10	392 (82.7)	82 (17.3)	
11 to 15	41 (83.7	8 (16.3)	
Religion			0.110
None	250 (82.0)	55 (18.0)	
Catholic	602 (81.5)	137 (18.5)	
Non-Catholic	229 (87.1)	34 (12.9)	
Personal mental health history			0.163
No	972 (82.2)	210 (17.8)	
Yes	109 (87.2)	16 12.8()	
Family mental health history			0.189
No	915 (82.1)	199 (17.9)	
Yes	166 (86.0)	27 (14.0)	
Categorized BMI			0.139
Underweight	235 (85.1)	41 (14.9)	
Normal	667 (81.0)	157 (19.1)	
Overweight	142 (85.5)	24 (14.5)	
Obesity	37 (90.2)	4 (9.8)	
Closeness with family members			0.003
Infrequent	356 (88.1)	48 (11.9)	
Frequent	474 (80.3)	117 (19.8)	
Very frequent	251 (80.5)	61 (19.6)	
Closeness with friends			<0.001
Infrequent	285 (90.8)	29 (9.2)	
Frequent	497 (80.9)	117 (19.1)	
Very frequent	299 (78.9)	80 (21.1)	
Academic performance			<0.001
Very poor	26 (89.7)	3 (10.3)	
Poor	44 (93.6)	3 (6.4)	
Fair	454 (86.6)	70 (13.4)	
Good	441 (81.2)	102 (18.8)	
Very good	116 (70.7)	48 (29.3)	
Failed a course during school years			0.007
No	569 (80.1)	141 (19.9)	
Yes	512 (85.8)	85 (14.2)	
In a romantic relationship			0.889
No	406 (82.2)	86 (17.5)	
Yes	675 (82.5)	140 (17.2)	
Cigarette use			0.019
No	1008 (82.1)	220 (17.9)	
Yes	73 (92.4)	6 (7.6)	
Alcohol use			0.697
Never	841 (82.3)	181 (17.7)	
Monthly (2 to 4 times)	208 (84.6)	38 (15.5)	
2 to 4 times per week	32 (82.1)	7 (18.0)	
Sought mental health support			
No	848 (82.1)	185 (17.9)	0.252
Yes	233 (85.0)	41 (15.0)	
Daily internet use			0.343
1 to 5 hours	658 (81.5)	149 (18.5)	
6 to 10 hours	249 (84.1)	47 (15.9)	
11 to 15 hours	174 (85.3)	30 (14.7)	
Daily television use			0.234
1 to 5 hours	993 (82.3)	214 (17.7)	
6 to 10 hours	64 (90.1	7 (9.9)	
11 to 15 hours	24 (82.8)	5 (17.2)	
Family member hospitalized due to COVID-19			0.101
No	548 (84.4)	101 (15.6)	
Yes	533 (81.0)	125 (19.0)	
Family member deceased due to COVID-19			0.191
No	611 (83.9)	117 (16.1)	
Yes	470 (81.2)	109 (18.8)	

*p-value calculated using the Chi-square test of independence.

### Factors associated with resilience in multiple regression analysis

**Figure 2:**
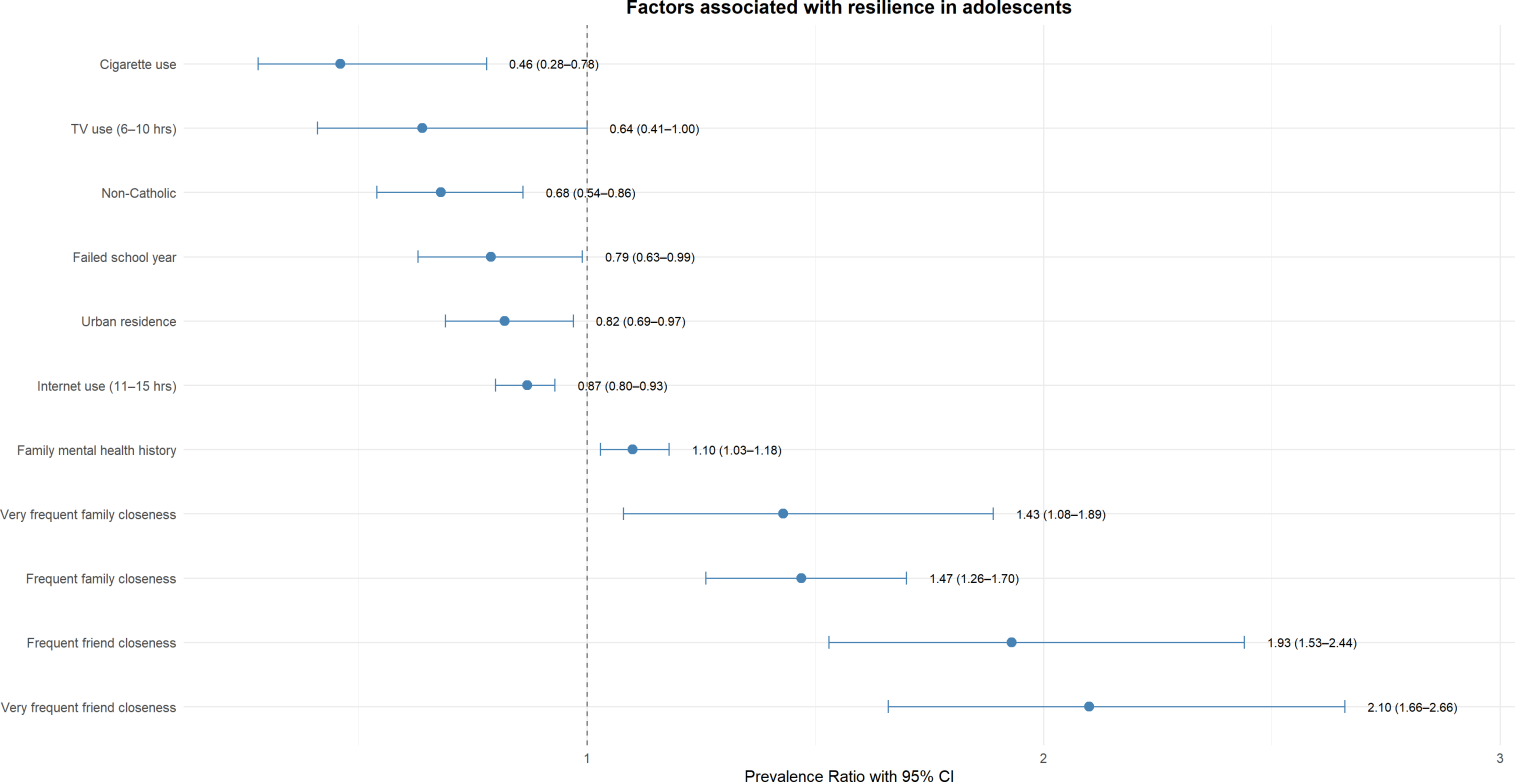
Factors associated with resilience in multiple regression analysis.

In multiple regression analysis, residing in urban areas was associated with an 18% decrease in the prevalence of high resilience compared to rural areas (PR: 0.82). Belonging to a religion other than Catholic reduced the prevalence of high resilience by 32% (PR: 0.68). Adolescents who reported frequent closeness with friends had almost twice the prevalence of high resilience compared to those with less social contact (PR: 1.93). Similarly, adolescents with very frequent closeness with friends had 2.10 times higher prevalence of high resilience (PR: 2.10). Regarding the relationship with family, adolescents with frequent and very frequent closeness with relatives had 47% (PR: 1.47) and 43% (PR: 1.43) higher prevalence of high resilience, respectively. Adolescents who had failed any course had 22% lower prevalence of resilience compared to those who did not fail (PR: 0.78). In addition, adolescents who used cigarettes presented a 54% decrease in the prevalence of resilience compared to non-smokers (PR: 0.46). On the other hand, the frequency of internet use of more than 11 hours per day was associated with a 13% reduction in the prevalence of resilience (PR: 0.87). Likewise, television use between 6 to 10 hours per day decreased the prevalence of resilience by 36% (PR: 0.64) (**Table 3**, **Figure 2**).

**Table 3:** Factors associated with resilience in simple and multiple regression analysis among adolescents.

Characteristics	Resilience					
	Simple regression			Multiple regression*		
	PR	95% CI	p**	PR	95% CI	p**
Adolescent developmental stage						
Early	Ref.					
Middle	1.20	0.89-1.62	0.236			
Late	0.99	0.65-1.51	0.973			
Sex						
Male	Ref.					
Female	1.27	0.97-1.67	0.078			
Type of school						
Public	Ref.					
Private	1.02	0.65-1.61	0.923			
School grade						
First	Ref.					
Second	1.02	0.58-1.78	0.958			
Third	1.17	0.97-1.41	0.101			
Fourth	1.39	0.66-2.92	0.386			
Fifth	1.08	0.56-2.08	0.823			
Residence area						
Rural	Ref.			Ref.		
Urban	0.83	0.70-0.99	0.037	0.82	0.69-0.97	**0.022**
Peri-urban	1.14	0.80-1.64	0.469	1.20	0.84-1.71	0.323
Number of family members (categorized)						
1 to 5	Ref.					
6 to 10	1.00	0.85-1.78	0.947			
11 to 15	0.94	0.64-1.38	0.756			
Religion						
None	Ref.			Ref.		
Catholic	1.03	0.79-1.34	0.839	0.96	0.74-1.24	0.753
Non-Catholic	0.72	0.53-0.97	0.029	0.68	0.54-0.86	**0.001**
Personal mental health history						
No	Ref.					
Yes	0.72	0.32-1.60	0.422			
Family mental health history						
No	Ref.					
Yes	0.78	0.52-1.18	0.246			
Categorized BMI						
Underweight	Ref.					
Normal	1.28	0.84-1.95	0.243			
Overweight	0.97	0.76-1.25	0.834			
Obesity	0.66	0.21-2.01	0.461			
Closeness with family members						
Infrequent	Ref.			Ref.		
Frequent	1.67	1.45-1.92	**<0.001**	1.47	1.26-1.70	**<0.001**
Very frequent	1.64	1.16-2.34	**0.006**	1.43	1.08-1.89	**0.013**
Closeness with friends						
Infrequent	Ref.			Ref.		
Frequent	2.06	1.54-2.77	**<0.001**	1.93	1.53-2.44	**<0.001**
Very frequent	2.29	1.94-2.69	**<0.001**	2.10	1.66-2.66	**<0.001**
Academic performance						
Very poor	Ref.					
Poor	0.62	0.21-1.779	0.373			
Fair	1.29	0.46-3.61	0.626			
Good	1.82	0.88-3.75	0.107			
Very good	2.83	0.96-8.33	0.059			
Failed a course during school years						
No	Ref.			Ref.		
Yes	0.72	0.59-0.87	**0.001**	0.79	0.63-0.99	**0.039**
In a romantic relationship						
No	Ref.					
Yes	0.98	0.84-1.16	0.834			
Cigarette use						
No	Ref.			Ref.		
Yes	0.42	0.24-0.73	**0.002**	0.46	0.28-0.78	**0.004**
Alcohol use						
Never	Ref.					
Monthly (2 to 4 times)	0.87	0.64-1.19	0.390			
2 to 4 times per week	1.01	0.76-1.36	0.928			
Sought mental health support						
No	Ref.					
Yes	0.83	0.70-1.00	0.052			
Daily internet use						
1 to 5 hours	Ref.			Ref.		
6 to 10 hours	0.86	0.59-1.26	0.442	0.83	0.57-1.22	0.349
11 to 15 hours	0.80	0.73-0.87	**<0.001**	0.87	0.80-0.93	**<0.001**
Daily television use						
1 to 5 hours	Ref.			Ref.		
6 to 10 hours	0.56	0.38-0.82	**0.003**	0.64	0.41-1.0	**0.049**
11 to 15 hours	0.97	0.53-1.77	0.927	1.39	0.65-2.97	0.392
Family member hospitalized due to COVID-19						
No	Ref.					
Yes	1.22	0.97-1.54	0.095			
Family member deceased due to COVID-19						
No	Ref.					
Yes	1.17	0.90-1.53	0.247			

*Adjusted for covariates of interest.

**p-values obtained with Generalized Linear Models (GLM), Poisson family, log link function, robust variance, school as a cluster.Bold values indicate statistically significant associations (p < 0.05).

## Discussion

### Prevalence of resilience

During the post-pandemic transition period, a minority of adolescents in our study were classified as having high resilience. This is consistent with post-pandemic findings from high school students and university students in China during the COVID-19 period (52, 53). This is contrary to the study conducted among first year students of Stomatology in Cuba in a pre-pandemic context (54). It also differs from findings in Colombian schoolchildren in a pre-pandemic setting (55). Similarly, a study in Chilean adolescents reported high levels of resilience (56).

The high frequency of low prevalence found in our study could be explained by a complex combination of interrelated factors. Family and socioeconomic difficulties, such as parental divorce and economic stress, are intertwined with mental health problems, risk behaviors such as depression and substance abuse, as well as experiences of abuse and discrimination. These challenges are compounded by the pandemic context, which has generated uncertainty, anxiety, and significant changes in daily life. In addition, individual characteristics, such as level of intelligence and self-control, also influence adolescents’ coping abilities. Taken together, these complex interactions among family, social, mental health, and contextual factors contribute to the difficulty many adolescents face in developing resilience during this challenging period (57–60).

The considerable variability in reported prevalence of high resilience across international studies warrants discussion (11–15). Global estimates among adolescents have ranged widely. Such discrepancies likely reflect differences in measurement tools, cut-off thresholds, study designs (e.g., cross-sectional vs. longitudinal), and the socio-cultural context in which resilience is assessed. For instance, while some studies adopt quartile or percentile-based classifications, others use fixed thresholds such as ≥30 on the CD-RISC-10 scale (48–50). Moreover, cultural norms regarding emotional expression, access to psychological support, and prevailing stressors (e.g., poverty, violence, or pandemic-related trauma) may influence how resilience manifests and is reported. In the Peruvian context, where adolescents often experience academic pressures, economic uncertainty, and post-pandemic psychological sequelae, the relatively low prevalence of high resilience observed in our study (17.3%) may reflect both contextual vulnerability and cultural framing of adversity. These findings underscore the need for culturally sensitive interpretations and locally validated instruments when evaluating psychological resilience in diverse populations.

### Factors associated with resilience

Residing in urban areas was associated with a lower prevalence of high resilience among adolescents. This finding aligns with studies conducted in China, where urban students showed lower resilience scores than their rural counterparts (61). Conversely, a study in Kenya found the opposite—living in urban areas was linked to lower resilience (62). This association could be explained by community disunity, since, in urban areas, there tends to be less social cohesion and solidarity among inhabitants, which disfavors mutual support in times of difficulty (63); connection with nature, since direct contact with nature can contribute to people’s emotional and mental well-being (64); and the pace of life, since in urban areas it tends to be more dynamic and stressful, which can make it difficult for people to better cope with the challenges they face (65). In the Peruvian sociocultural context, this association may reflect unique challenges faced by urban adolescents. Urban environments in Peru are often characterized by higher exposure to stressors such as noise, pollution, overcrowding (66), delinquency, and organized crime (67). These conditions may compromise adolescents’ sense of safety and increase chronic stress. Moreover, urban areas may present weaker community ties and reduced neighborhood cohesion, limiting the informal social support networks that are critical for emotional resilience (68). In contrast to rural areas, where interdependence and collective caregiving are more common, urban adolescents may experience greater social fragmentation and isolation. Additionally, the accelerated and competitive pace of urban life, along with increased exposure to social media and performance-based pressures, may further undermine the development of stable coping mechanisms. Finally, reduced exposure to natural environments in cities—known to contribute to psychological well-being (69) could also play a role in weakening resilience (70). These complex sociocultural dynamics should be considered when designing urban youth mental health policies and interventions in Peru.

We found that having a non-Catholic religion was associated with a lower prevalence of high resilience. Notably, many resilience-focused studies in adolescents do not include religion as a variable (71–74). However, some studies in other populations have explored this relationship. For instance, Camara GF et al. reported higher resilience scores among Brazilian medical students with non-Catholic affiliations (75), and Edara IR et al. found a positive correlation between religiosity and resilience in Taiwanese university students (76). By contrast, Gan SKE et al. found no significant association in Singaporean university students (77). This result should be interpreted cautiously, as it may reflect sociocultural dynamics rather than a direct effect of religious belief. In Peru—where Catholicism is the dominant, historically institutionalized religion (78), non-Catholic adolescents may face social marginalization, underrepresentation in school events, or limited access to culturally aligned emotional support (79). They may also lack structured spiritual spaces or mentoring resources more accessible within Catholic institutions, especially in public schools (80). Thus, the association may reflect social inclusion disparities rather than inherent differences between religions (81).

Adolescents who had failed a school course showed a lower prevalence of high resilience. This finding aligns with studies that report a positive association between academic performance and resilience among high school students in Spain (73) and China (82, 83), although some studies found no significant association (84). This association could be explained by the fact that low academic performance may be an indicator of difficulties in the school environment, such as lack of motivation, learning difficulties or problems adapting to the educational environment (85). Another explanation is that the negative impact of poor academic performance extends beyond the school setting and may have consequences in the personal and social lives of adolescents. Experiences of failure can generate negative emotions such as shame or discouragement, hindering the ability to face and overcome other challenges effectively (86). Likewise, the family environment and the expectations of parents and guardians can influence the perception of academic success or failure, which in turn can impact adolescents’ self-image and resilience. Lack of support or excessive pressure to perform well may contribute to feelings of stress and decreased resilience (83). Lack of support or excessive pressure to perform well may contribute to feelings of stress and decreased resilience (87).

Adolescents who maintained frequent or very frequent contact with their friends were more likely to exhibit higher levels of resilience. This is similar to that reported in Ukrainian children, where they found that a child-friendly school environment fosters a higher level of resilience (88). Similar to that described in Chinese schoolchildren, where he found an inverse relationship between relationships with friends and the level of resilience (84). A study conducted in Korean children found a relationship between both variables (89). This is contrary to that described in children from China, where he found an indirect association between resilience and frequency to friends (90). The protective role of friendship may be explained by the emotional and affective support peers provide, which fosters self-esteem, confidence, and a sense of belonging (91). These elements create a safe environment for emotional expression and problem-solving. Furthermore, ongoing interaction with friends promotes the development of social competencies such as empathy, communication, and conflict resolution that are essential for coping with stress and adversity (92).

Adolescents who reported cigarette smoking had a lower prevalence of high resilience. This is similar with that reported in Iranian students where they found that resilience was negatively associated with water pipe smoking (93). This is contrary with that mentioned in Pennsylvania adults, where they suggest that smoking is not associated with the level of resilience (94). The negative association observed in our study may reflect the link between smoking and maladaptive coping strategies, emotional dysregulation, or increased vulnerability to stress, all of which can undermine the development of resilience during adolescence (95).

Adolescents who reported using the internet between 11 to 15 hours per day reduced the prevalence of high resilience. This is similar to that reported in Tunisian students, where they found that resilience is negatively associated with internet addiction (96). A study conducted in medical students found a strong negative correlation between internet use and resilience (97). In Chinese adolescents found a negative association between internet addiction and resilience (98). Similarly, during the COVID-19 pandemic, social distancing and increased screen time were associated with poorer mental health outcomes and reduced coping capacity (99). This association could be because prolonged Internet use could displace activities that promote resilience, such as physical activity, face-to-face social interactions, and participation in extracurricular activities (100). This could negatively affect the development of coping and adaptive skills that are critical for resilience (101). Another explanation is that excessive use of electronic devices, especially before bedtime, may interfere with sleep quality (102). Sleep deprivation or interrupted sleep can affect mood, concentration, and the ability to handle stress, which influences resilience (103).

Adolescents who reported watching television between 6 to 10 hours per day reduced the prevalence of high level of resilience. This is supported by a study in US children and adolescents which found that the lower the television exposure (less than or equal to 2 hours per day) the higher the level of resilience (104). Another study conducted in the United States reported that resilience is associated with less time spent in sedentary behaviors such as watching television (105). Likewise, research among Chinese children and adolescents found that those who watched TV more than 2 hours daily had more probability of low resilience compared to those who watched less than 1 hour daily (106). These associations may reflect the displacement of cognitively or socially enriching activities such as physical exercise, face-to-face interaction, or academic engagement by excessive screen use (107). The consistent evidence from diverse populations suggests that long hours of television viewing may hinder the development of emotional and behavioral resources essential for resilience (108, 109).

Beyond statistical significance, the magnitude of several associations observed in this study indicates their practical importance in adolescent mental health. For example, adolescents with very frequent closeness to friends had more prevalence of high resilience, highlighting the strong protective role of peer relationships. Likewise, frequent closeness with family members increased the prevalence of high resilience, reinforcing the central role of familial support. In contrast, cigarette use was associated with a reduction in resilience, and daily internet use exceeding 11 hours reduced resilience. These findings suggest that promoting supportive social environments and addressing modifiable behaviors—such as digital overuse and substance use—could have a substantial impact in strengthening adolescents’ capacity to cope with adversity. Therefore, effect sizes from our multivariate analysis underscore not only statistical relevance but also actionable targets for public health interventions.

Although our study included COVID-19–related variables such as the hospitalization or death of a family member, these were not found to be significantly associated with resilience in the multivariate analysis and were therefore not included in the final model. However, it is important to acknowledge that the COVID-19 pandemic likely served as a shared background stressor that shaped adolescents’ emotional context, routines, and support systems. The absence of a statistically significant effect does not rule out the possibility of more complex, moderated, or time-sensitive impacts, especially in vulnerable subgroups. Future research should consider stratified analyses or qualitative designs to better understand how direct and indirect pandemic-related stressors influenced the development of resilience, particularly in adolescents who experienced personal loss or prolonged isolation.

### Relevance of mental health findings

Adolescence is a unique stage of human development; however, the physical, emotional, and social changes that occur during this period, along with factors such as poverty, abuse, or exposure to violence, can increase the vulnerability of adolescents to present mental health problems (110). Resilience is one of the main factors affecting human health and in the case of adolescents it allows them to cope with the changes they experience during this stage, preventing the development of mental health problems (14). It is an important protective mechanism for coping with adversity (111) and serves as protection against depression and sleep problems (112). Consequently, it is important to address resilience factors in educational and therapeutic settings for adolescents so that they can better adapt to various events in their daily routines (31). Our findings provide essential information that can guide health managers in making evidence-based decisions to improve the emotional well-being and mental health of adolescents. We recommend implementing mentoring and psychological counseling programs targeting all students, with special attention to those with low academic performance and students who do not profess the Catholic faith (46). These interventions have the potential to strengthen resilience and promote effective coping strategies. Likewise, awareness campaigns on the appropriate use of technology and the risks associated with smoking are suggested in order to promote healthy behaviors among adolescents. The organization of inclusive fraternization events for all students can contribute to creating a school environment that promotes kindness, respect and mutual trust, which are essential for emotional well-being and the development of positive relationships (113). Additionally, it is proposed to provide specific counseling for students coming from urban areas, with the objective of facilitating their adaptation to the school environment and providing them with tools to face the challenges that may arise. These activities are designed to involve both students and their parents or caregivers, recognizing the importance of family support in the process of strengthening the resilience and mental health of adolescents.

Our findings offer practical guidance for designing targeted interventions within the school setting to strengthen resilience in adolescents. Schools should consider implementing peer mentoring programs that foster supportive friendships and social connection, particularly given the strong protective association observed between frequent closeness with friends and resilience. Additionally, given the negative associations with excessive internet and television use, digital wellness programs aimed at promoting healthy screen habits could serve as preventive strategies to enhance emotional regulation and attention.

The integration of psychosocial support teams, including school psychologists, counselors, and trained teachers can help identify students with low resilience and provide tailored interventions, especially for those with academic difficulties or limited family support. Schools in urban areas may require specific programming that addresses the elevated stress and reduced cohesion observed in these environments. Finally, culturally sensitive approaches should be adopted when addressing students from non-Catholic backgrounds, ensuring inclusivity and representation in institutional support services. These policy-oriented actions could have a meaningful impact on improving adolescents’ capacity to cope with adversity.

### Limitations and strengths

We recognize the limitations of our study. First, the cross-sectional design does not allow us to establish causality. Second, nonresponse bias, due to possible variations in the levels of motivation to voluntarily participate in the study among adolescents. Third, information bias due to the fact that other factors influencing adolescent resilience have not been assessed, such as family functioning (114), teacher-student relationship (115), self-esteem (116), and social support (117). Fourth, selection bias due to the fact that all study participants completed the questionnaire voluntarily. Fifth, the use of non-probability (convenience) sampling limits the generalizability of our findings to the broader adolescent population. Although this method facilitated efficient data collection from a large school-based sample in northern Peru, it may not fully capture the heterogeneity of adolescents from other regions or educational settings. To address this limitation in future research, we recommend the adoption of more representative sampling strategies, such as stratified random sampling or multistage cluster sampling, which would enhance external validity and the potential applicability of the findings to national populations. Sixth, the study assessed gender using only binary categories (male and female), which limits the inclusivity and comprehensiveness of our findings. This approach may not capture the experiences and resilience levels of adolescents with non-binary or diverse gender identities. Seventh, many of the variables analyzed—such as substance use, academic performance, and interpersonal relationships—were based on self-reported data. This may have introduced response bias due to social desirability or recall limitations, particularly for sensitive topics like smoking or academic failure. Future studies should consider using triangulation strategies, such as incorporating academic records, behavioral logs, or reports from parents and teachers, to enhance the validity of the findings. Eighth, potential interaction effects between key variables—such as gender × internet use or academic performance × family support—were not examined in this study. While our main objective was to identify direct associations with resilience, future research should explore interaction terms to uncover potential moderating effects and provide a more understanding of how combined factors influence resilience in adolescents. Ninth, due to the cross-sectional nature of our study, we were unable to observe how resilience evolves over time or in response to changing personal and environmental conditions. Resilience is not a static trait but a dynamic process that can develop or decline during adolescence. Therefore, we recommend that future research employs longitudinal designs to track resilience trajectories and identify temporal patterns and predictors. Moreover, mixed-methods studies that incorporate qualitative insights could enrich the understanding of how adolescents interpret and respond to adversity, and how these experiences shape their resilience development over time.

However, our study presents several methodological strengths that reinforce the validity and robustness of its findings. First, we used a validated and widely recognized questionnaire for the assessment of resilience, which guarantees reliability and accuracy in the measurement of this variable. A key strength of this study is the high internal consistency of the CD-RISC-10 in our sample, supporting the reliability of the resilience scores obtained. In addition, the sample was of considerable size, which made it possible to achieve adequate statistical power, reducing the probability of type II errors and improving the ability to detect significant associations. Likewise, this study addressed a wide variety of sociodemographic, academic, family and behavioral factors, providing a comprehensive analysis of the determinants of resilience in adolescents. Unlike previous studies, variables explored little in the literature were included, such as internet and television use, substance use, mental health history, and experience with the COVID-19 pandemic, which broadens the understanding of factors that may influence resilience in this age group. Another key strength was the application of robust statistical modeling, using a cluster analysis approach at the educational institution level, reducing bias arising from the hierarchical structure of the data. Finally, the study was conducted in a post-pandemic context, providing up-to-date evidence on resilience in adolescents following a global event with significant mental health repercussions. This provides relevant information for the formulation of intervention strategies in the educational and health care settings to strengthen resilience in this population.

## Conclusions

This study estimated the prevalence and associated factors of resilience among adolescents from five educational institutions in northern Peru. Only 17.3% demonstrated a high level of resilience, suggesting that many students struggle to adapt to and cope with adversity. Several protective factors were identified, including frequent or very frequent contact with family and friends, highlighting the importance of social support in fostering resilience. Additionally, adolescents with good academic performance and no history of course failure reported higher resilience levels, indicating that academic success may facilitate adaptive coping. Conversely, risk factors such as living in urban areas, identifying with a non-Catholic religion, cigarette use, and excessive internet and television exposure were linked to lower resilience. These findings highlight the need to address both contextual and behavioral factors in interventions targeting adolescent resilience.

From a public health and educational standpoint, our findings support the implementation of structured interventions, such as mentoring, psychological counseling, and initiatives promoting healthy lifestyle habits and digital hygiene. Strategies that strengthen social connections and encourage extracurricular participation may also support the development of emotional and cognitive coping skills. Finally, this study provides relevant post-pandemic evidence on adolescent resilience. Future research should consider longitudinal designs to monitor resilience over time and explore additional determinants, including family functioning, teacher-student relationships, and exposure to traumatic experiences.

## Data Availability

The original contributions presented in the study are included in the article/supplementary material. Further inquiries can be directed to the corresponding author.
